# A Comparative Study on Deep Learning Models for COVID-19 Forecast

**DOI:** 10.3390/healthcare11172400

**Published:** 2023-08-26

**Authors:** Ziyuan Guo, Qingyi Lin, Xuhui Meng

**Affiliations:** 1 Xiangya School of Medicine, Central South University, Changsha 410008, China; 2 School of Mathematics and Statistics, Huazhong University of Science and Technology, Wuhan 430074, China

**Keywords:** COVID-19, infectious prediction, deep learning, deep neural network

## Abstract

The COVID-19 pandemic has led to a global health crisis with significant morbidity, mortality, and socioeconomic disruptions. Understanding and predicting the dynamics of COVID-19 are crucial for public health interventions, resource allocation, and policy decisions. By developing accurate models, informed public health strategies can be devised, resource allocation can be optimized, and virus transmission can be reduced. Various mathematical and computational models have been developed to estimate transmission dynamics and forecast the pandemic’s trajectories. However, the evolving nature of COVID-19 demands innovative approaches to enhance prediction accuracy. The machine learning technique, particularly the deep neural networks (DNNs), offers promising solutions by leveraging diverse data sources to improve prevalence predictions. In this study, three typical DNNs, including the Long Short-Term Memory (LSTM) network, Physics-informed Neural Network (PINN), and Deep Operator Network (DeepONet), are employed to model and forecast COVID-19 spread. The training and testing data used in this work are the global COVID-19 cases in the year of 2021 from the Center for Systems Science and Engineering (CSSE) at Johns Hopkins University. A seven-day moving average as well as the normalization techniques are employed to stabilize the training of deep learning models. We systematically investigate the effect of the number of training data on the predicted accuracy as well as the capability of long-term forecast in each model. Based on the relative $L_{2}$ errors between the predictions from deep learning models and the reference solutions, the DeepONet, which is capable of learning hidden physics given the training data, outperforms the other two approaches in all test cases, making it a reliable tool for accurate forecasting the dynamics of COVID-19.

## 1. Introduction

The coronavirus disease 2019 (COVID-19) pandemic [1], caused by the severe acute respiratory syndrome coronavirus-2 (SARS-CoV-2), has resulted in an unprecedented global health crisis with substantial morbidity and mortality rates, as well as profound socioeconomic disruptions [2,3,4,5,6]. Since its initial emergence in Wuhan, China in December 2019, COVID-19 has swept across the globe, with over 200 million confirmed cases and millions of fatalities reported worldwide by 2021. Unfortunately, many variants of the COVID-19 virus have been recently detected worldwide, and the disease still appears in multiple waves. Therefore, this pandemic has underscored the urgency of understanding and predicting the dynamics of COVID-19 to inform public health interventions, resource allocation, and policy decisions in the pursuit of mitigating its impact on societies [7,8].

The SARS-CoV-2 virus is primarily transmitted through respiratory droplets, aerosols, and contaminated surfaces, with an incubation period ranging between 2 and 14 days. The broad spectrum of COVID-19 clinical manifestations includes asymptomatic and mild cases, as well as severe respiratory distress and multi-organ failure, leading to hospitalizations and fatalities. The elderly, individuals with underlying health conditions, and immunocompromised patients represent the most vulnerable populations to severe outcomes [9,10,11,12]. The number of infections needs to be monitored and predicted, which is critical for developing prevention and care plans [6,8,13,14].

Predicting the prevalence of COVID-19 infections is essential to inform targeted and timely interventions, optimize resource allocation, and enable effective communication to the public. These predictions can guide decision-makers in implementing strategies such as social distancing measures, mass testing, contact tracing, and vaccination campaigns, ultimately reducing the spread of the virus and saving lives [7,15,16]. Furthermore, accurate predictions can facilitate the evaluation and adjustment of implemented interventions in response to changing epidemic conditions, allowing for more adaptable and efficient management of the pandemic.

Due to the urgent need for accurate prediction models to inform public health interventions, resource allocation, and policy decisions, a number of mathematical and computational models have been developed to estimate the transmission dynamics and forecast the short- and long-term trajectories of the pandemic [17,18,19]. However, the complex and rapidly evolving nature of the COVID-19 pandemic requires innovative approaches to enhance the accuracy and applicability of these predictions [8]. Machine learning (ML), a subset of artificial intelligence (AI) that enables computers to learn from and analyze data [20,21], offers a promising solution to this challenge by incorporating large-scale, diverse data sources and providing data-driven insights to improve COVID-19 prevalence predictions. In the realm of data prediction, the deep neural networks (DNNs) have demonstrated unparalleled effectiveness in various domains, ranging from natural language processing and image recognition to financial forecasting and medical diagnostics [22,23,24,25]. These advancements have, in turn, fueled the development of novel applications and techniques that can address complex challenges, such as predicting the spread of infectious diseases, including the COVID-19 pandemic.

Considering that the COVID-19 cases are time series data similar as those in financial forecasting and medical diagnostics, the DNNs have also been utilized for the COVID-19 predictions. In particular, the long-short-term-memory (LSTM) networks [26] as well as its variants [27] are widely employed for COVID-19 forecast due to its capability for providing accurate long-term predictions. Just to name a few related examples, Chimmula et al., employed the LSTM model to predict the daily confirmed cases in various countries, demonstrating accurate predictions on a short-term basis [28]. Furthermore, Yang et al., utilized the LSTM in conjunction with other machine learning techniques to model the spatial-temporal patterns of the pandemic, providing a useful tool for guiding public health interventions [29].

It is worth mentioning that the LSTM is a purely data-driven method, which is risky for very long-term predictions [30]. The recently developed physics-informed neural networks (PINNs) [31,32], which can encode the physical laws in DNNs, is able to achieve better accuracy especially in extrapolations compared to the purely data-driven deep learning models since the predictions follow the physics after training. The PINNs have also been applied to investigations of the spread of COVID-19 [33,34]. As reported in [33,35], the PINN can provide more accurate results in long-term predictions than the purely data-driven deep learning model by encoding the partially known physics in the DNNs. We note that the accuracy of PINNs strongly depends on the the physical laws or mathematical models encoded in the DNNs. We may not be able to obtain good results if the mathematical model is misspecified in PINNs. Developing accurate mathematical models for describing the dynamics of COVID-19 remains challenging, which limits the further improvements to the accuracy of PINNs for COVID-19 forecast.

Most recently, the deep operator networks (DeepONets) have been proposed to learn the hidden physics represented by the training data [36,37]. Theoretical work has shown that the DeepONet is a universal approximator to any nonlinear operator [36]. Unlike PINNs, DeepONets do not require to explicitly encode the physical laws or mathematical model in the DNNs. The inherent physics can be directly learned given sufficient data. Some studies have leveraged this property to predict time series using DeepONet, such as forecasting the state-of-charge of the solar system [38], which shown that DeepONet outperforms the LSTM in these specific cases. To the best of our knowledge, the effectiveness of DeepONet for COVID-19 forecast has not been justified yet.

Despite various deep learning approaches have been developed and employed for COVID-19 forecast, how to select the most suitable DNN models for predicting the prevalence of COVID-19 infections remains an open question. In the present study, we will conduct a comprehensive comparative study on the performance of different deep learning models for COVID-19 forecast. Particularly, we will test the performance of three types of deep learning models, i.e., the data-driven, physics-informed and the operator learning models. The findings will help future researchers choose most suitable DNN models for predicting how COVID-19 would transmit.

The rest of this work is organized as follows: In Section 2, we discuss the detailed problem setup as well as the deep learning models employed in this study. The numerical results are presented in Section 3, and the present study is consluded in Section 4.

## 2. Methods

### 2.1. Problem Setup

In this work, we utilize the COVID-19 global cases throughout the year of 2021, which are obtained from the Center for Systems Science and Engineering (CSSE) at Johns Hopkins University [39]. On 1 January 2021, at the beginning of our analysis window, the worldwide count of newly reported cases had contributed to an overall tally of 84,332,767 cases. On 31 December 2021, at the end of our window, the total case count had surged to 288,631,129 cases. In summary, the reporting window revealed three pronounced waves. At the beginning, the daily new cases exhibited an upward and subsequent downward oscillation within the first month, reaching its trough around mid-February 2021 with a daily addition of approximately 280,000 cases. The second wave of infections peaked around May 2021, reaching about 900,000 new cases. It then decreased significantly, reach the bottom with around 30,000 daily additions by late June 2021. Then, about four months after the previous high point, in mid-August, it surged again and reached another peak. This was followed by a decline, reaching another low point about four months after the previous nadir.

**Figure 1:**
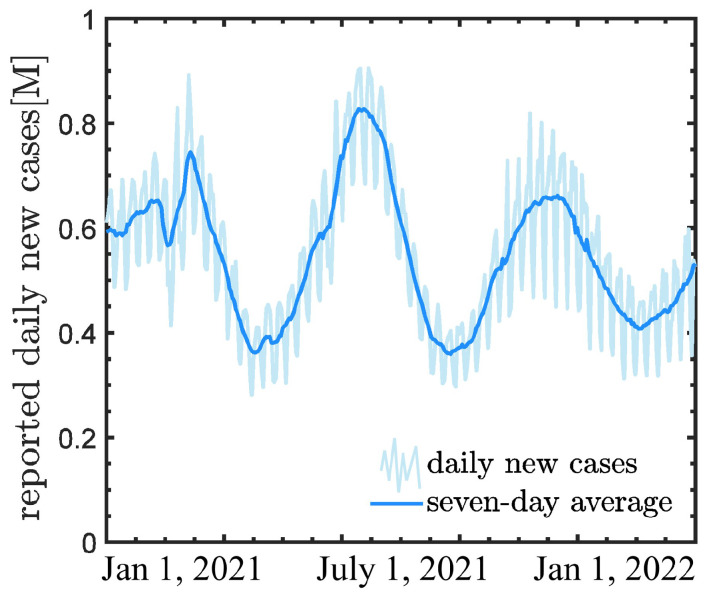
Normalized daily new COVID-19 cases worldwide in 2021.

As observed, there are significant fluctuations in the raw data. we then utilize a sliding window to generate smooth training and testing data, with the seven-day moving average, which is illustrated in Figure 1. Further, we normalize the new cases as follows to make the training of deep learning models more stable.

(1) $$n=\frac{n-n_{min}}{n_{max}-n_{min}}\times 0.3,$$

where $n_{min}$ and $n_{max}$ are the minimum and maximum values of the total initial data, respectively. We note that the data used in the current study has also been employed in [33]. Also, the seven-day moving average is also utilized to stabilize the training of neural networks.

In particular, we will employ three different deep learning models for forecasting of the new cases, i.e., LSTM, PINN, and DeepONet. To be more specific, the LSTM, PINNs, and DeepONets are utilized as the representative for the data-driven, physics-informed and the operator learning deep learning models, respectively. As mentioned, the LSTM is a widely used deep learning model for time series forecasting; the PINN is capable of leveraging both data and the partially known physics, which generally leads to more accurate extrapolations in time series forecasting; and the DeepONet which is capable of learning the hidden operators represented by the training data, which is also promising in providing accurate predictions for time series forecasting. The objective of the present study is to conduct a comprehensive comparison on the computational accuracy of the three aforementioned deep learning models for COVID-19 forecasting. Specifically, we will consider the following cases:

- Case A: we study the effect of the size of sliding windows in LSTM and DeepONet, laying the foundation for selecting window size in subsequent work;
- Case B: We employ the daily new COVID-19 cases in the first 240 days of 2021 as training data, and forecast the new COVID-19 cases in the remaining 120 days, which is referred to as the standard case in this study for comparing the results of these three methods;
- Case C: The effect of of different numbers of training data on the predicted accuracy is investigated. Particularly, we use the first 220, 240, and 260 days’ new COVID-19 cases as the training data, respectively; and predict the new cases in the remaining days of 2021;
- Case D: This case focuses on the performance of long-term extrapolation in different methods. We employ the COVID-19 cases in the first 220 days of 2021 as the training data, and predict the new cases in the following 180 days rather than 120 days in Case A.

In the present study, we use the relative $L_{2}$ error between the predictions and the reference solutions as the evaluation metric to evaluate the predicted accuracy of each model, which is defined as

(2) $$E=\frac{\sqrt{\sum_{j=1}^{N_{D}}{\left(\Phi \left(d_{j}\right)-\Phi_{NN}\left(d_{j}\right)\right)}^{2}}}{\sqrt{\sum_{j=1}^{N_{D}}\Phi {\left(d_{j}\right)}^{2}}},$$

where Φ is the public data for new infections of COVID-19, $\Phi_{NN}$ is the prediction results, and $N_{D}$ is the total number of days in the prediction window. A smaller value of *E* indicates better predicted accuracy. We note that the $L_{2}$ error has also been employed as a metric in previous studies [31,40,41,42,43].

### 2.2. Deep Learning Models

We present the details for the three deep learning models, i.e., LSTM, PINNs, and DeepONet, in this section.

#### 2.2.1. Long Short-Term Memory Model

**Figure 2:**
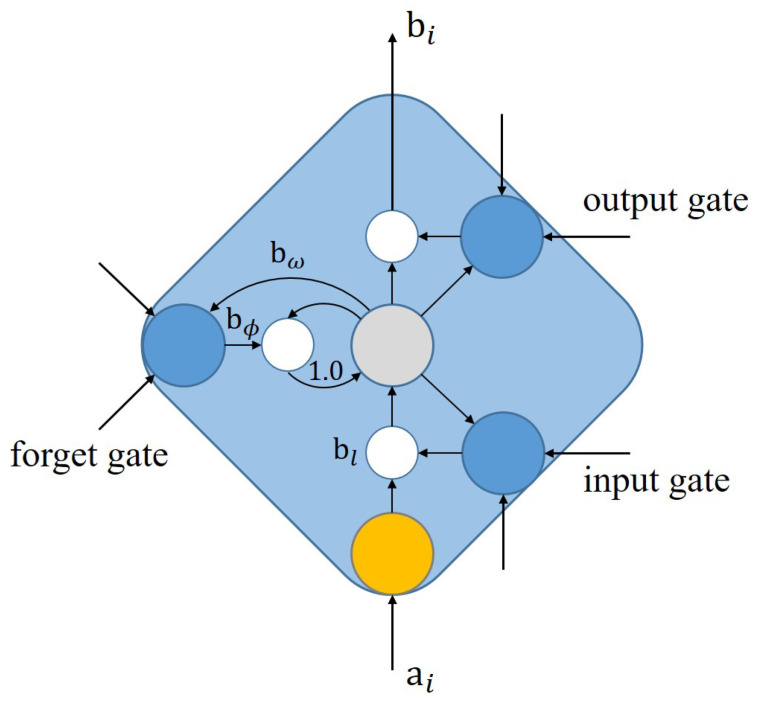
Schematic of LSTM memory cell with gating units.

The LSTM model is a type of recurrent neural network (RNN) architecture developed by Hochreiter and Schmidhuber in 1997 to address the vanishing gradient problem in traditional RNNs [26]. The key innovation of LSTM lies in its memory cell, which is designed to store long-term dependencies in sequences more effectively. This feature makes LSTM particularly suitable for a wide range of sequence-based tasks, such as natural resources and stock forecasting [25,44]. For a standard nerual network unit it only consists the input activation $a_{i}$ and the output activation $b_{i}$, while LSTM networks have gating units, including input, output, and forget gates, that enable selective retention and modification of information in the memory cell, seen in Figure 2. This selective information flow allows LSTM to be more robust to noise and irrelevant input features, while also handling variable-length input sequences [45]. The LSTM has three of these gates to protect and control the cell state:

(3) $$\begin{array}{cc} \; & f_{i}=\sigma \left(b_{l}\cdot a_{i}+bias_{f}\right),\\ \; & i_{i}=\sigma \left(b_{\omega }\cdot a_{i}+bias_{i}\right),\\ \; & C_{i}=tanh\left(b_{\Phi }\cdot a_{i}+bias_{C}\right),\\ \; & b_{i}=f_{i}*C_{i}+i_{i}*C_{i}. \end{array}$$

#### 2.2.2. Physics-Informed Neural Networks

In recent years, there has been a remarkable surge of interest in the application of the physics-informed neural networks (PINNs) for predictive modeling. PINNs combine the power of deep learning with the physical principles encoded in partial differential equations (PDEs) to solve complex problems in a data-driven manner. By incorporating physical knowledge into the network architecture, PINNs can effectively capture the underlying physical laws and learn accurate solutions even from limited or noisy data.

Considering parameterized and nonlinear partial differential equations of a general form:

(4) $$u_{t}+N\left[u;\lambda \right]=0,x\in \Omega ,t\in \left[0,T\right],$$

where u(t;x) denotes the solution, N[u;λ] is a nonlinear operator parameterized by λ, and Ω is a subset of $R^{D}$. The loss function for training the PINNs is expressed as follows:

(5) $$MSE=MSE_{u}+MSE_{f},$$

where

(6) $$\begin{array}{cc} \; & MSE_{u}=\frac{1}{N_{u}}\sum_{i=1}^{N_{u}}{\left|u\left(t_{u}^{i},x_{u}^{i};\lambda \right)-u^{i}\right|}^{2},\\ \; & MSE_{f}=\frac{1}{N_{f}}\sum_{i=1}^{N_{f}}{\left|f\left(t_{f}^{i},x_{f}^{i};\lambda \right)\right|}^{2}, \end{array}$$

with $f=u_{t}+N\left[u;\lambda \right]$ denoting the residual of the equation. Various studies have demonstrated the efficacy of PINNs across diverse fields, including fluid dynamics, material science, and biomedical engineering, which makes it a promising tool for advancing both fundamental scientific research and practical applications.

**Figure 3:**
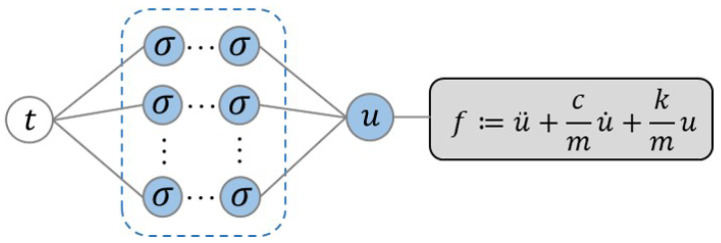
Schematic of PINNs. The governing equation is encoded into the DNN using automatic differentiation.

In this work, we employ the equation for modeling a damped harmonic oscillator as the governing equation for the description of the dynamics for COVID-19 following [33], which is expressed as follows:

(7) $$f:=\ddot{u}+\frac{c}{m}\dot{u}+\frac{k}{m}u=0,$$

where *u* is the number of infections, seen in Figure 3. In this case we fix the value of the mass to m=1, and the remaining parameter *k* and *c* are unknown in the equation, which will be determined given data.

#### 2.2.3. Deep Operator Networks

**Figure 4:**
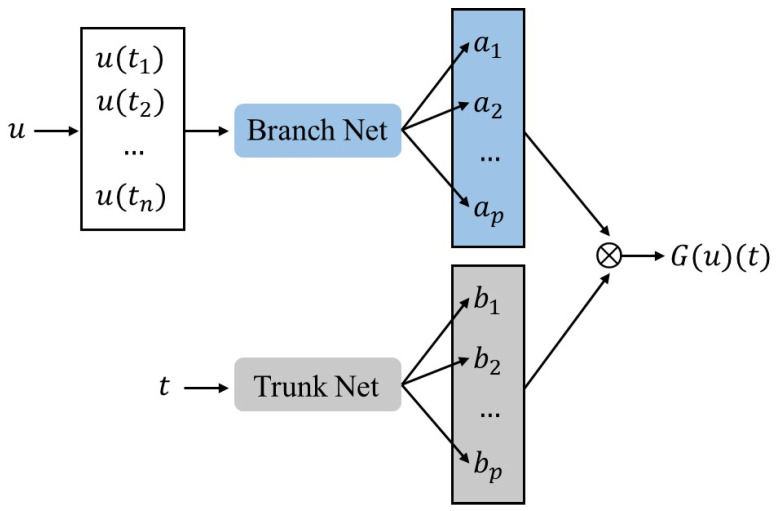
Schematic of DeepONet.

By leveraging the expressive power of deep neural networks, DeepONet can learn complex mappings between inputs and outputs, making it well-suited for solving high-dimensional and nonlinear PDEs [36,46]. The DeepONet is essentially a mapping between two function spaces. As shown in Figure 4, the DeepONet is composed of two sub-networks, i.e., the Branch Net (BN) and the Trunk Net (TN) (Figure 4). The input for BN is a function, which is represented by a set of discrete function values at certain locations, i.e., $t_{1},t_{2},\ldots ,t_{n}$, and the output of BN is a vector $\left[a_{1},a_{2},\ldots ,a_{p}\right]$. In addition, TN takes *t* as input and outputs a vector $\left[b_{1},b_{2},\ldots ,b_{p}\right]$. The output of the DeepONet is the inner product of these two vectors:

(8) $$u\left(t\right)=G\left(u\right)\left(t\right)=\sum_{k=1}^{p}a_{k}b_{k}.$$

**Figure 5:**
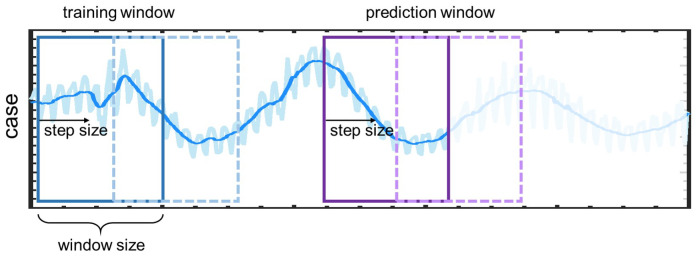
Schematic of the sliding window in LSTM and DeepONet. Blue line: training window, purple line: prediction window.

In LSTM and DeepONet, we use sliding windows based on the historical data to generate the training data [46,47]. As shown in Figure 5, the LSTM/DeepONet takes the data in the training window as input, and predict the new cases in the prediction window. Specifically, we refer to the number of data in the training/prediction window as window size. Also, the window size of the training and prediction window is the same, and the step size for sliding the window is kept as 1 in the present study.

## 3. Results and Discussion

In this section, we will conduct a comprehensive performance comparison among three different deep learning models, i.e., the LSTM, PINN, and DeepONet, on COVID-19 forecast. In the following computations, (1) the LSTM has 1 hidden layer with 100 neurons per layer, (2) for the DeepONet, the branch net has 2 hidden layers with 50 neurons per layer, and the trunk net also has 2 hidden layers with 50 neuron per layer. The number of dimensions for the output of the branch/trunk net is 120, and (3) in PINNs, the DNN has 2 hidden layers with 32 neurons per layer, which is the same as in [33]. In addition, the hyperbolic function is utilized as the activation functions in all deep learning models. In each method, we employ the mean-squared error (MSE) between the reference and the predictions from the deep learning models as the loss functions. The Adam optimizer with an initial learning rate $10^{-3}$ will be used in the training of all deep learning models. We note that all models are implemented using the deep learning framework Tensorflow.

### 3.1. Case A: Effect of the Size of Sliding Window in LSTM and DeepONet

**Figure 6:**
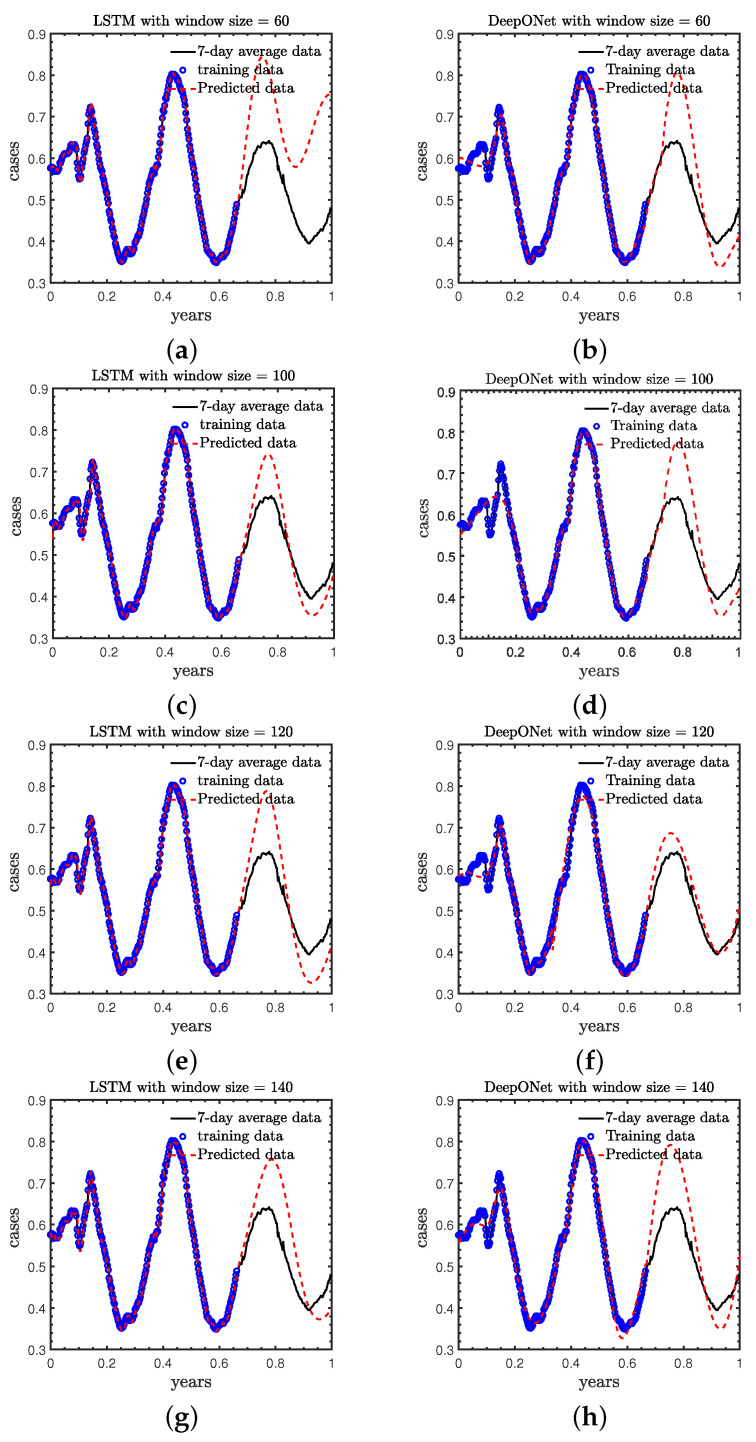
Predictions from LSTM and DeepONet with different sliding windows. LSTM: Left column (**a**,**c**,**e**,**g**); DeepONet: Right column (**b**,**d**,**f**,**h**).

We first study the effect of the size of sliding windows in LSTM and DeepONet on the predicted accuracy, laying the foundation for selecting window size in subsequent work. In this case the sliding windw size is set to be 60, 100, 120 and 140 for both LSTM and DeepONet with step sizes fixed at 1. We employ the daily new COVID-19 cases in the first 240 days of 2021 as training data, and forecast the new COVID-19 cases in the remaining 120 days. The results are shown in Figure 6 and Table 1.

As observed: (1) the LSTM obtains the most accurate predictions as the window size is 100, and (2) the DeepONet achieve the best accuracy as the window size is 120. In general, increasing the size of the slide window is able to provide more information on the dependency of the time series, which expects to improve the predicted accuracy. However, a larger prediction window requires the deep learning models to forecast longer time series, which may decrease the predicted accuracy of the deep learning models. Therefore, in the following study we set the size of the sliding window as 100 and 120 in LSTM and DeepONet, respectively.

### 3.2. Case B: Standard Case

**Figure 7:**
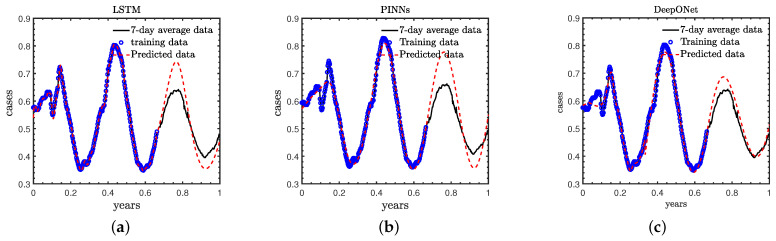
Predictions for the COVID-19 new cases using different deep learning models for Case A: (**a**) LSTM, (**b**) PINNs, (**c**) DeepONet. Blue circles: Training data, Black solid line: Reference, Red dashed line: Predictions.

In this subsection, we employ LSTM, PINNs and DeepONet to forecast the new COVID-19 infection cases and compare the results of these methods. We note that the PINN is equipped with the same physical model and similar data as used in [33], which is served as the baseline for the comparison here. The architectures as well as the training data of LSTM and DeepONet are the same as in Case A. It is observed in Figure 7 and Table 2 that: (1) the LSTM is able to fit the training data better than the PINN as well as DeepONet; (2) the predictions for the remaining 120 days from the LSTM and PINNs are quite similar. They share the same trends with the reference solutions, but the magnitudes are quite different from those in the reference solutions; and (3) the DeepONet is capable of providing quite accurate predictions for both the trends as well as the numbers of the new cases.

We would like to discuss here that: (1) the LSTM is a purely data-driven method, which is expected to obtain better results with more training data; (2) the PINN leverages both the data and the partially known physics. However, we may also need more data to identify the unknown physics in order to achieve better accuracy. We will show more details on the effect of the number of training data on the predicted accuracy for the LSTM and PINNs in the next section; and (3) the DeepONet is capable of learning the hidden physics given data in the first 240 days, and hence provides the most accurate predictions among the three different deep learning models.

### 3.3. Case C: Effect of Number of Training Data

As mentioned in the previous section, we expect the LSTM as well as PINNs to achieve better accuracy with more training data. We now conduct a detailed study to address the effect of the number of training data in the LSTM and PINNs. Specifically, we test the cases with the new COVID-19 cases in the first 220, 240, and 260 days as the training data, and forecast the cases in the remaining days of 2021.

**Figure 8:**
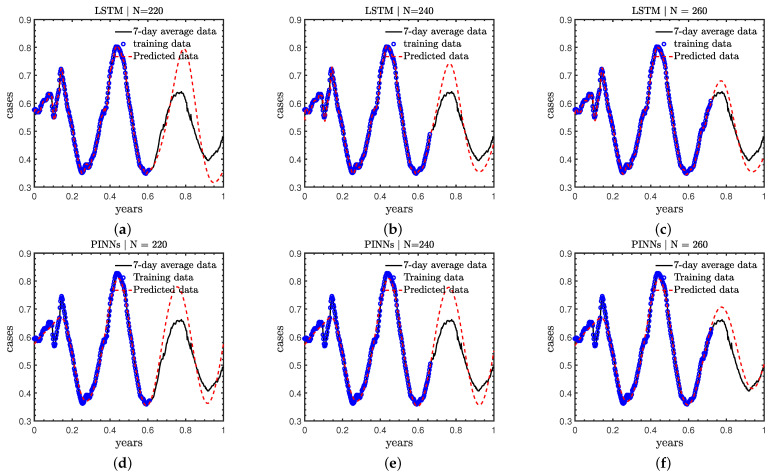
Predicted COVID-19 new cases with different training numbers for LSTM [Top row, (**a**–**c**)] and PINNs [Bottom row, (**d**–**f**)].

As displayed in Figure 8 and Table 3: (1) both the LSTM and PINN achieve better accuracy as we increase the number of the training data; (2) the PINN is able to provide the trends more accurately than the LSTM in all test cases. For instance, the locations of the peaks and valleys in the prediction window from the PINNs agree better with the reference solutions than the LSTM, especially for the first case in which we have less training data; (3) both the PINN and LSTM can obtain good accuracy in the case we use the data in the first 260 days; and (4) the PINN has better accuracy than the LSTM for the predictions at t>0.9 years in these specific cases, which demonstrates that the learned physics can improve the accuracy for extrapolation.

### 3.4. Case D: Performance for Long-Term Forecast

**Figure 9:**
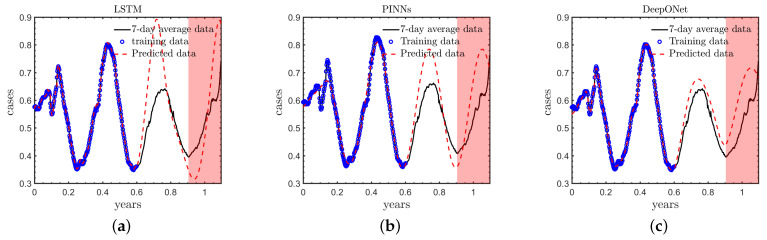
Long-term predictions for COVID-19 new cases using different deep learning models. (**a**) LSTM, (**b**) PINNs, (**c**) DeepONet.

To further investigate the capability of different deep learning models for long-term forecast for this specific task, we reduce the number of training data while extending the model’s prediction time compared to the standard case in Case B. We selected the first 220 days new cases as the training data and generated predictions for the following 180 days. So the total number of days in this case is 400 days. The red shade indicates the extended prediction segment, seen in Figure 9. As illustrated in Figure 9 and Table 4: (1) all models fit the training data well; (2) the DeepONet is the most accurate among the three deep learning approaches for the long-term forecast, which is smilar as in Case B; and (3) the computational errors in PINNs are smaller than those in LSTM, which indicates that the inclusion of the partially known physics is able to improve the predicted accuracy, especially for extrapolation. We note that the results here are similar as those in Case B and C, which will not be discussed in detail again.

## 4. Conclusions

In recent years the application of deep learning to disease prediction has received increasing interest, especially since the outbreak of COVID-19. A number of deep-learning models have been developed for forecasting the spreed of COVID-19 with considerable success. This work present a comparative study on the prediction performance of three well-established deep learning models, i.e., LSTM, PINN, and DeepONet). Particularly, (1) the LSTM is a purely data-driven approach, (2) the PINN is able to encode the partially known physics in the DNNs given training data, and (3) the DeepONet is capable of learning the hidden physics represented by the training data. The effects of number of training data as well as the size of sliding window on the predicted accuracy in the deep learning approaches are investigated.The numerical experiments conducted in the present study show that the DeepONet outperforms the other two models, and provides a reliable tool for accurate forecasting the dynamics of COVID-19.

We would like to discuss that deep learning models often lack interpretability. Although some theoretical work has been developed to improve the interpretability of complex models like PINN as well as DeepONet [32,36,37], more rigorous analyses on these model, e.g., the effect of the neural architecture on the computational accuracy, are not available yet. In this work, we confirm the effectiveness of the PINN as well as DeepONet for COVID-19 forecast using the validation data in the prediction window empirically. Furthermore, techniques like the neural architecture search, self-adaptive weights for different loss terms, may be helpful to improve the predicted accuracy for the deep learning models employed in this work. The deep learning models employed in the present study are used to predict the new cases given historical data, which can be useful for the preparation for a possible second wave. However, they are not able to tell the detailed reasons that cause the new cases to grow or decrease. A possible way for this issue is to develop mathematical models to describe the effect of every important factor that affects the spreading of the COVID-19 and encode them in PINNs. We leave the study of these important topics in the future.

## Figures and Tables

**Table 1:** The prediction error *E* from LSTM and DeepONet with different sliding windows.

*E*	Size = 60	Size = 100	Size = 120	Size = 140
LSTM	37.1%	10.3%	15.7%	16.3%
DeepONet	17.4%	16.1%	6.2%	17.2%

**Table 2:** The prediction errors (*E*) of the different deep learning models.

	LSTM	PINNs	DeepONet
*E*	6.2%	13.0%	10.3%

**Table 3:** The computational errors (*E*) for LSTM and PINNs.

*E*	N=220	N=240	N=260
LSTM	18.7%	10.3%	8.2%
PINNs	12.9%	13.0%	8.5%

**Table 4:** The long-term prediction errors (*E*) of different deep learning models.

	LSTM	PINNs	DeepONet
*E*	25.4%	17.6%	14.7%

## Data Availability

Data available on request due to restrictions e.g., privacy or ethical. The data presented in this study are available on request from the corresponding author. The data are not publicly available due to the restrictions.
